# Taxonomy and Molecular Phylogeny of Two New Urostylid Ciliates (Protozoa: Ciliophora) From Chinese Wetlands and Establishment of a New Genus

**DOI:** 10.3389/fmicb.2021.707954

**Published:** 2021-07-30

**Authors:** Wenya Song, Tengyue Zhang, Jingyi Dong, Xiaotian Luo, William A. Bourland, Yurui Wang

**Affiliations:** ^1^Laboratory of Protozoological Biodiversity and Evolution in Wetland, College of Life Sciences, Shaanxi Normal University, Xi’an, China; ^2^Institute of Evolution and Marine Biodiversity, Ocean University of China, Qingdao, China; ^3^Department of Zoology, Comenius University in Bratislava, Bratislava, Slovakia; ^4^Key Laboratory of Aquatic Biodiversity and Conservation of Chinese Academy of Sciences, Institute of Hydrobiology, Chinese Academy of Sciences, Wuhan, China; ^5^Department of Zoology, Faculty of Science, Charles University, Prague, Czechia

**Keywords:** ciliated protists, core urostylids, integrative taxonomy, phylogeny, 18S rRNA gene

## Abstract

Hypotrich ciliates with evolutionary novelties are continually being discovered, challenging the current taxonomic system and attracting increased attention. In the present work, two new urostylid ciliates, *Heterobakuella bergeri* gen. nov., sp. nov. and *Anteholosticha perezuzae* sp. nov., isolated from Chinese wetland samples, were identified based on morphology and 18S rRNA gene sequences. *Heterobakuella* gen. nov. is defined by three frontal cirri, single buccal cirrus, one parabuccal cirrus, midventral complex composed of cirral pairs and one cirral row, one left and two right marginal cirral rows, transverse and pretransverse cirri present, caudal and frontoterminal cirri absent. *Heterobakuella* can be easily distinguished from the morphologically most similar genus, *Apobakuella*, mainly by the single buccal cirrus (vs. one buccal cirral row) and one parabuccal cirrus (vs. several parabuccal cirral rows originated from different anlagen). Phylogenetic analyses show that *H. bergeri* branches within the clade formed by *Bergeriella ovata*, *Monocoronella carnea*, *Anteholosticha gracilis*, and *Neourostylopsis* spp., rather than the clade represented by *Apobakuella*. The other species, *A. perezuzae*, is mainly characterized by a distinctly slender body shape with an average length:width ratio about 7, distinctively shaped biconcave and greenish cortical granules, as well as one or two pretransverse cirri. Phylogenetic analyses indicate the genus *Anteholosticha* is non-monophyletic.

## Introduction

Hypotrichia Stein, 1859, is a large group of ciliated protists with extremely diverse morphologic and morphogenetic characters, with worldwide distribution in diverse habitats, including marine and fresh waters, and even desert soils (Berger, 1999, 2006, 2008, 2011; Foissner, 2016; Hu et al., 2019; Jung and Berger, 2019; Kaur et al., 2019; Kim and Min, 2019; Wang et al., 2020a,b; Chen et al., 2021; Luo et al., 2021; Ma et al., 2021; Song et al., 2021; Vd’ačný and Foissner, 2021; Wang et al., 2021). Urostylids, with more than 200 described species, are one of the largest groups in the subclass Hypotrichia (Berger, 2006). Studies from the past few decades have revealed that this group is even more diverse than previously thought, and many new species are likely awaiting discovery (Berger, 2006; Jiang et al., 2013; Jo et al., 2015; Kim et al., 2016; Song and Shao, 2017; Chen et al., 2018, 2020; Zhu et al., 2019; Shao et al., 2020; Xu et al., 2020; Zhang et al., 2020a). In addition, some classifications at the family and genus levels within this group (e.g., Bakuellidae Jankowski, 1979 and *Anteholosticha* Berger, 2003), and their systematic relationships, remain confusing (for reviews, see Berger, 2006; Huang et al., 2014; Lyu et al., 2015, 2018; Moon et al., 2020; Zhang et al., 2020b).

According to Berger (2006), some urostylid taxa characterized by having three frontal cirri and a midventral complex composed of cirral pairs in the anterior portion and at least one cirral row in the posterior portion were assigned to the family Bakuellidae. Accordingly, at least 10 genera, i.e., *Apobakuella* Li et al., 2011, *Australothrix* Blatterer and Foissner, 1988, *Bakuella* Agamaliev and Alekperov, 1976, *Birojimia* Berger and Foissner, 1989, *Holostichides* Foissner, 1987, *Metaurostylopsis* Song et al., 2001, *Monourostylopsis* Song et al., 2020, *Neobakuella* Li et al., 2011, *Parabirojimia* Hu et al., 2002, and *Paragastrostyla* Hemberger, 1985 could be assigned to this family (Berger, 2006; Li et al., 2011; Jiang et al., 2013; Song et al., 2020). However, molecular studies showed that the family Bakuellidae was not monophyletic, and some of its genera (e.g., *Australothrix*, *Birojimia*, and *Parabirojimia*) had phylogenetic incongruence between the morphological and molecular data, thus its classification scheme was questioned and some newly established families (e.g., Hemicycliostylidae Lyu et al., 2018 and Parabirojimidae Dai and Xu, 2011) were proposed later (Lynn, 2008; Dai and Xu, 2011; Foissner, 2016; Lyu et al., 2018). In addition, new bakuellid-like taxa with evolutionary novelties are regularly being discovered, which also renders the classification of the bakuellid-like taxa difficult in practice.

The genus *Anteholosticha* was established by Berger (2003) for some species previously assigned to *Holosticha* sensu Borror (1972), which lack distinct autapomorphies (e.g., anterior end of left marginal cirral row curved rightwards, adoral zone bipartite, buccal cirrus distinctly ahead of paroral membrane). Many studies have shown that *Anteholosticha* is extremely divergent (Berger, 2006, 2008; Park et al., 2012, 2013; Huang et al., 2014; Lyu et al., 2015; Zhao et al., 2015; Chen et al., 2018, 2020; Jung et al., 2021). Hence, Huang et al. (2014) assigned three species, *Anteholosticha warreni* (Song and Wilbert, 1997) Berger, 2003, *A. scutellum* (Cohn, 1866) Berger, 2003, and *A. petzi* Shao et al., 2011, each with a roughly U-shaped pattern of transverse cirri and forming a clade distinctly separated from other *Anteholosticha* groups in molecular trees, to the newly erected genus *Arcuseries*. To date, more than 40 nominal species of *Anteholosticha* have been reported, although many of them are poorly documented, with only simple morphologic descriptions and without molecular data. All these factors hamper our understanding of the systematics and taxonomy of this genus (Berger, 2006; Fan et al., 2016; Chen et al., 2018, 2020).

More studies, based on morphologic, molecular, and ecologic data, are thus urgently needed to tackle these problems. In this work, two novel wetland urostylid ciliates, *Heterobakuella bergeri* gen. nov., sp. nov. and *Anteholosticha perezuzae* sp. nov., are reported and their phylogenetic positions are discussed.

## Materials and Methods

### Sample Collection

**FIGURE 1:**
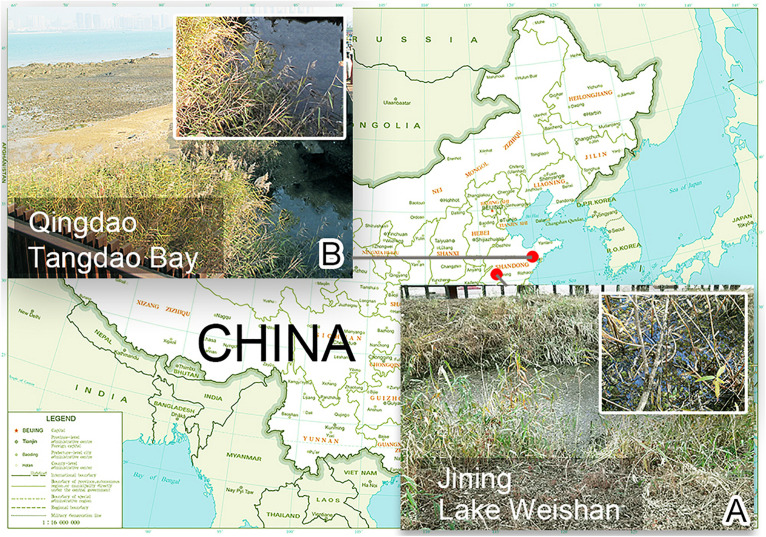
Locations of sampling sites. **(A)** Surroundings of the sampling site of Lake Weishan, the city of Jining, where *Heterobakuella bergeri* gen. nov., sp. nov. was collected. **(B)** Photograph of Tangdao Bay, the city of Qingdao, where *Anteholosticha perezuzae* sp. nov. was collected.

*Heterobakuella bergeri* gen. nov., sp. nov. was collected on November 12, 2019 from a small brook that flows into the Weishan Lake Wetland, administrated by the city of Jining, Shandong Province, China (34°46′14″N, 117°12′56″E) (Figure 1A), when the water temperature was 15°C. *Anteholosticha perezuzae* sp. nov. was isolated from the sample collected from a brackish water stream that originates from a small lake wetland near Tangdao Bay, Qingdao, China (35°56′18″N, 120°12′44″E) (Figure 1B) on March 27, 2017, where the water temperature was 15°C and salinity was 8‰. In each case, sampling water (up to 20 cm deep) was first stirred, then about 300 ml of water including sediments, mud, and rotten plants was collected and transferred to our laboratory. Next, the sample was divided into several aliquots that were used to establish raw cultures in Petri dishes (diameter = 9 cm). All raw-culture samples were maintained at room temperature (about 23°C). Because the two species we studied could be easily distinguished from other species present in the same Petri dish by body shape and color, the confusion with other species was avoided.

### Morphological Observation

Live specimens were observed under the microscope (Olympus BX53; Olympus Corporation, Tokyo, Japan) with magnifications ranging from 100× to 1000×. Protargol impregnation was performed to reveal ciliary patterns and nuclear apparatus (Wilbert, 1975). Drawings were based on free-hand sketches with the help of drawing devices. Classification and terminology mainly follow Berger (2006).

### DNA Extraction, PCR, and Sequencing

Five cells of each species were isolated from Petri dishes and washed several times using filtered and sterilized site water. Using micropipettes, cells were transferred into three 1.5-ml eppendorf tubes, two tubes with one cell each and the third with three cells. Genomic DNA was extracted using the DNeasy Blood and Tissue Kit (Qiagen, Germantown, MD, United States) according to the manufacturer’s protocol with 25% of the suggested reagent volumes as described by Lu et al. (2020). The 18S rRNA genes of *Heterobakuella bergeri* gen. nov., sp. nov. and *Anteholosticha perezuzae* sp. nov. were amplified with the same forward primer 82F (5′-GAAACTGCGAATGGCTC-3′) (Jerome et al., 1996) but different reverse primers, 5.8SR (5′-TACTGATATGCTTAAGTTCAGCGG-3′) (Wang J. et al., 2017) for *H. bergeri* gen. nov., sp. nov. and 18SR (5′-TGATCCTTCTGCAGGTTCACCTAC-3′) (Medlin et al., 1988) for *A. perezuzae* sp. nov. To minimize amplification errors during PCR, we used Q5 Hot Start High-Fidelity DNA Polymerase (New England BioLabs, United States) as recommended by Wang C. et al. (2017). The thermocycler program was set according to Bai et al. (2020). The PCR products were sequenced bidirectionally in the Tsingke Biological Technology Company (Qingdao, China) using the PCR primers and three internal primers, i.e., Pro + B (5′-GGTTAAAAAGCTCGTAGT-3′), 900F (5′-CGATCAGATACCGTCCTAGT-3′), and 900R (5′-ACTAGGACGGTATCTGATCG-3′) for both species (Wang J. et al., 2017).

### Phylogenetic Analyses

To infer the phylogenetic position of the two newly obtained species, we downloaded 18S rRNA gene sequences of 77 other ciliates from the National Center for Biotechnology Information (NCBI) database^1^, including 62 “core urostylids,” seven oxytrichids, three *Arcuseries* species, as well as the outgroup taxa composed of two *Uncinata* and three *Holosticha* species. GenBank accession numbers are shown in Figure 6. After combining all of the sequences, the alignment was carried out with the MUSCLE algorithm on the webserver GUIDANCE 2^2^ with default parameters (Sela et al., 2015). The primer sequences were manually trimmed using BioEdit v.7.0 (Hall, 1999). The maximum likelihood (ML) analysis was done using RAxML-HPC2 (XSEDE v.8.2.12) on the CIPRES Science Gateway server^3^ with 1,000 bootstrap replicates and the GTRGAMMA model of nucleotide substitution (Stamatakis, 2014). Bayesian inference (BI) analysis was carried out using MrBayes (Ronquist et al., 2012) on CIPRES Science Gateway (XSEDE v.3.2.6), with the GTR + I + G nucleotide substitution model selected under Akaike Information Criterion (AIC) by jModelTest 2 (Darriba et al., 2012). Four Markov chain Monte Carlo (MCMC) simulations were run for 1,000,000 generations with the first 2,500 sampled trees discarded as burn-in. Convergence was assessed using RWTY (Warren et al., 2017). SeaView v.4.6.1 (Gouy et al., 2010) and MEGA 6.0 (Tamura et al., 2013) were used to visualize the tree topologies.

**FIGURE 2:**
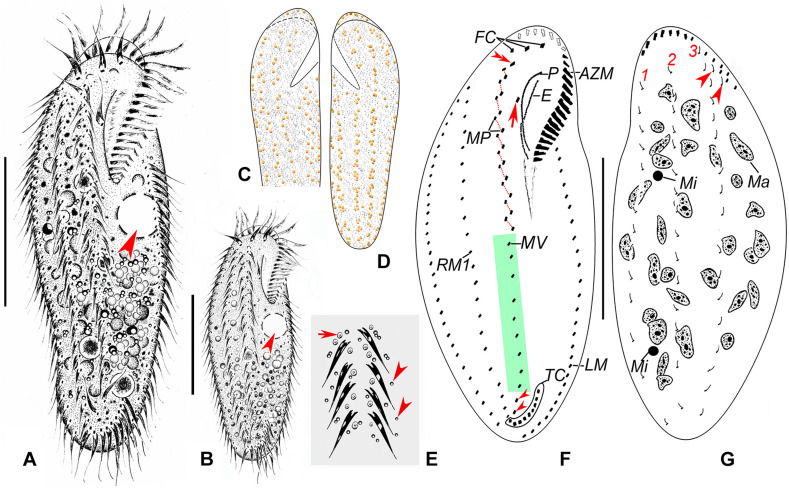
Morphology and infraciliature of *Heterobakuella bergeri* gen. nov., sp. nov. from life **(A–E)** and after protargol impregnation **(F,G)**. **(A,B)** Ventral views of a typical individual **(A)** and a different cell **(B)**, showing the body shape. Arrows depict the contractile vacuole. **(C–E)** Arrangement of cortical granules on ventral **(C,E)** and dorsal **(D)** sides, arrow and arrowheads in **(E)** indicate details of cortical granules and globules, respectively. **(F,G)** Ventral **(F)** and dorsal **(G)** views of the holotype specimen, showing infraciliature and nuclear apparatus. In **(F)**, arrow shows buccal cirrus, arrowheads mark pretransverse cirri, and double-arrowhead indicates parabuccal cirrus. Arrowheads in **(G)** indicate two additional dorsal bristles. AZM, adoral zone of membranelles; E, endoral membrane; FC, frontal cirri; LM, left marginal cirral row; Ma, macronuclear nodules; Mi, micronuclei; MP, midventral cirral pairs; MV, midventral cirral row; P, paroral membrane; RM1, right marginal cirral row 1; TC, transverse cirri; 1–3, dorsal kineties 1–3. Scale bars = 50 μm.

**FIGURE 3:**
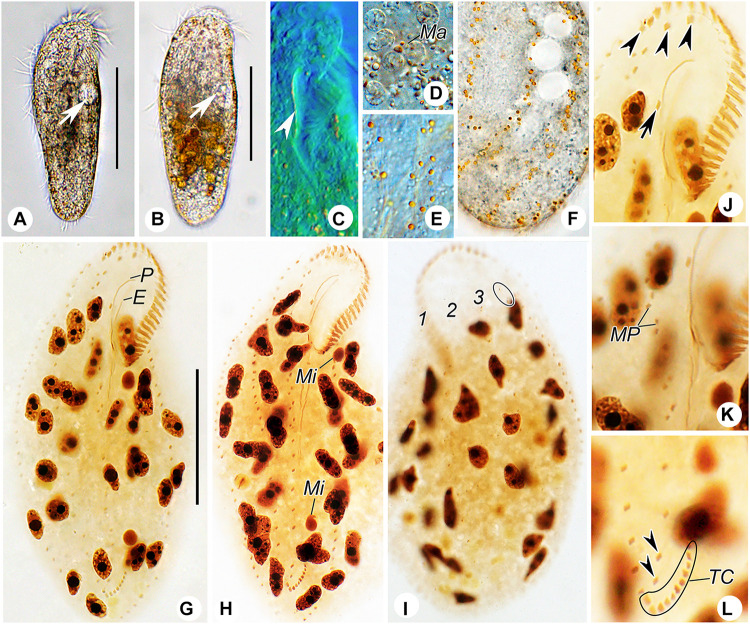
Photomicrographs of *Heterobakuella bergeri* gen. nov., sp. nov. from life **(A–F)** and after protargol impregnation **(G–L)**. **(A,B)** Ventral views of two representative specimens; arrows indicate the contractile vacuole. **(C)** Ventral view to show the undulating membranes. **(D)** Macronuclear nodules. **(E,F)** Ventral **(E)** and dorsal **(F)** views to show the arrangement of cortical granules. **(G,H)** Ventral overviews of the holotype **(G)** and a paratype **(H)** specimen, showing the ciliary pattern. **(I)** Dorsal view of a paratype specimen, to demonstrate dorsal kineties and the nuclear apparatus. Circle marks two additional dorsal bristles. **(J)** Ventral view, to show three frontal cirri (arrowheads) and buccal cirrus (arrow). **(K)** Ventral view, showing the midventral cirral pairs. **(L)** Ventral view, to show two pretransverse cirri (arrowheads) and transverse cirri. E, endoral membrane; Ma, macronuclear nodules; Mi, micronuclei; MP, midventral cirral pairs; P, paroral membrane; TC, transverse cirri; 1–3, dorsal kineties 1–3. Scale bars = 50 μm.

**FIGURE 4:**
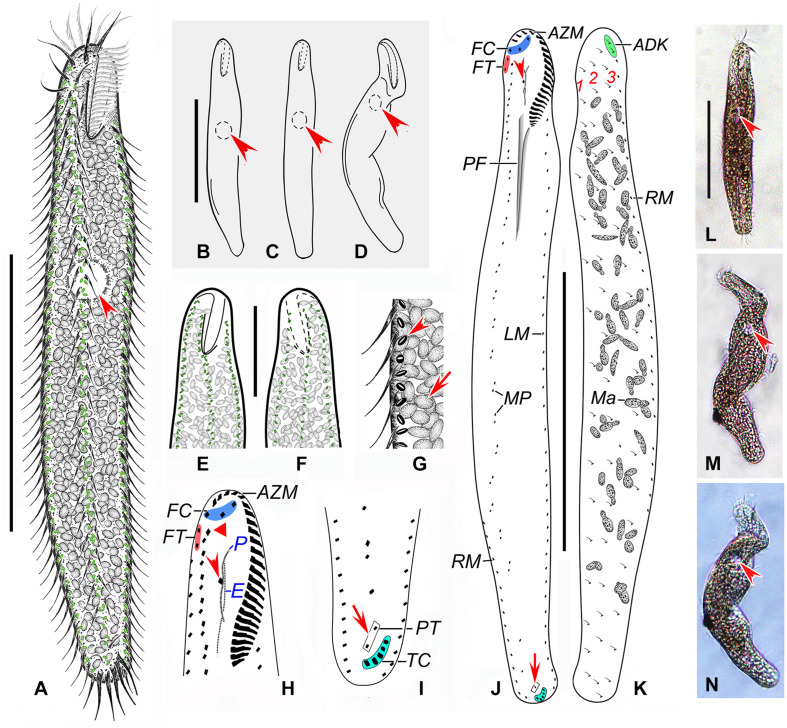
*Anteholosticha perezuzae* sp. nov. from life **(A–G,L–N)** and after protargol impregnation **(H–K)**. **(A–D)** Overviews, showing the different body shapes and flexibility, arrowheads mark the contractile vacuole. **(E–G)** Ventral **(E,G)** and dorsal **(F)** views of the surface, showing the arrangement of slightly biconcave-shaped cortical granules (arrowhead) and packed ellipsoidal structures, likely mitochondria (arrow). **(H)** Ventral view of anterior part of cell, showing buccal cirrus (arrowhead) and parabuccal cirrus (red triangle). **(I)** Ventral posterior view, showing pretransverse cirri (arrow) and transverse cirri (light-blue part). **(J,K)** Ventral **(J)** and dorsal **(K)** views of the holotype specimen, showing the ciliary pattern and the nuclear apparatus; arrowhead marks buccal cirrus and arrow indicates the pretransverse cirrus. **(L–N)** Overviews of different specimens; arrows indicate contractile vacuole. ADK, additional dorsal bristles at anterior end of the right marginal cirral row; AZM, adoral zone of membranelles; E, endoral membrane; FC, frontal cirri; FT, frontoterminal cirri; LM, left marginal cirral row; Ma, macronuclear nodules; MP, midventral cirral pairs; P, paroral membrane; PF, pharyngeal fibers; PT, pretransverse cirri; RM, right marginal cirral row; TC, transverse cirri; 1–3, dorsal kineties 1–3. Scale bars = 110 μm **(A–D,J–N)**, 70 μm **(E,F)**.

**FIGURE 5:**
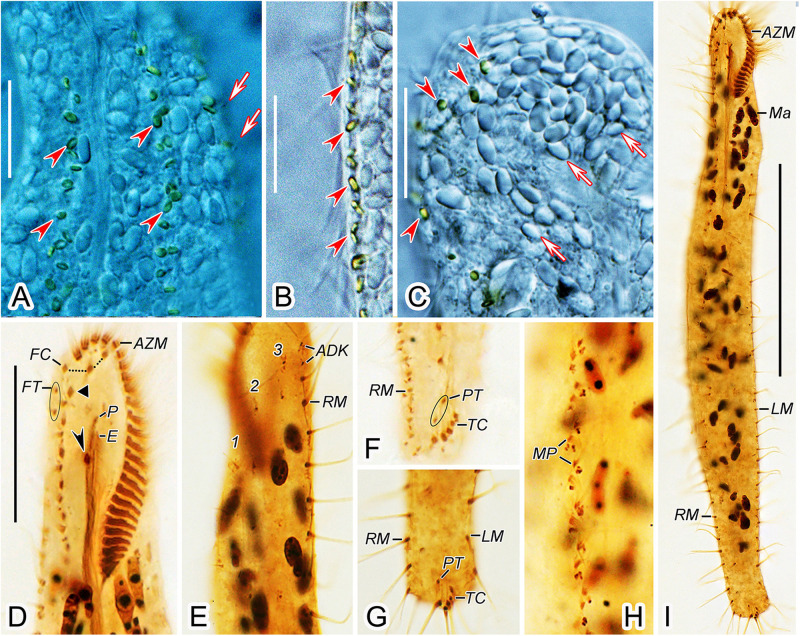
*Anteholosticha perezuzae* sp. nov. from life **(A–C)** and after protargol impregnation **(D–I)**. **(A–C)** Ventral **(A,B)** and dorsal **(C)** views of the surface; arrowheads mark cortical granules. Arrows in **(A)** represent dorsal cilia and arrows in **(C)** point probable mitochondria. **(D,E)** Ventral **(D)** and dorsal **(E)** views of the anterior body regions; arrowhead shows the buccal cirrus, black triangle indicates the parabuccal cirrus. **(F,G)** Ventral views of the posterior body region, showing marginal cirral rows, pretransverse cirri, and transverse cirri. **(H)** Midventral cirral pairs. **(I)** Ventral view of the holotype specimen. ADK, additional dorsal bristles at anterior end of the right marginal cirral row; AZM, adoral zone of membranelles; E, endoral membrane; FC, frontal cirri; FT, frontoterminal cirri; LM, left marginal cirral row; Ma, macronuclear nodules; MP, midventral cirral pairs; P, paroral membrane; PT, pretransverse cirri; RM, right marginal cirral row; TC, transverse cirri; 1–3, dorsal kineties 1–3. Scale bars = 10 μm **(A–C)**, 25 μm **(D–H)**, 100 μm **(I)**.

**FIGURE 6:**
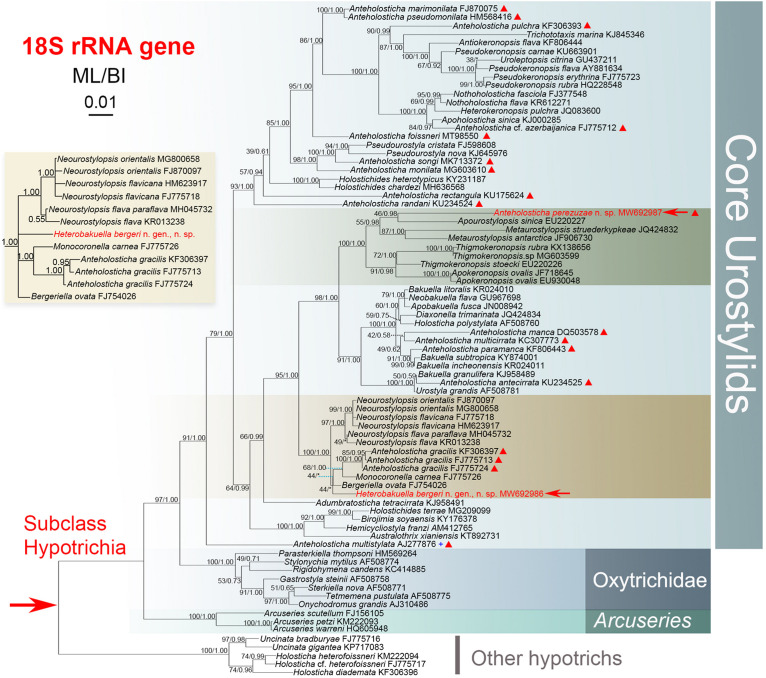
Maximum-likelihood (ML) tree inferred from 18S rRNA gene sequences, showing the systematic positions of *Heterobakuella bergeri* gen. nov., sp. nov. and *Anteholosticha perezuzae* sp. nov. (indicated in red). An inserted branch of *Heterobakuella bergeri* gen. nov., sp. nov. in Bayesian inference (BI), showing the systematic position of *H. bergeri*. Numbers near branches denote bootstrap values for ML and posterior probabilities for BI. Asterisks (*) indicate the disagreement between ML and BI trees and triangles indicate *Anteholosticha* species. The population of *Anteholosticha multistylata* marked with the plus (+) symbol was from NCBI without published morphological data. GenBank accession numbers are provided after species names. All branches are drawn to scale. The scale bar represents one substitution per 100 nucleotides.

**FIGURE 7:**
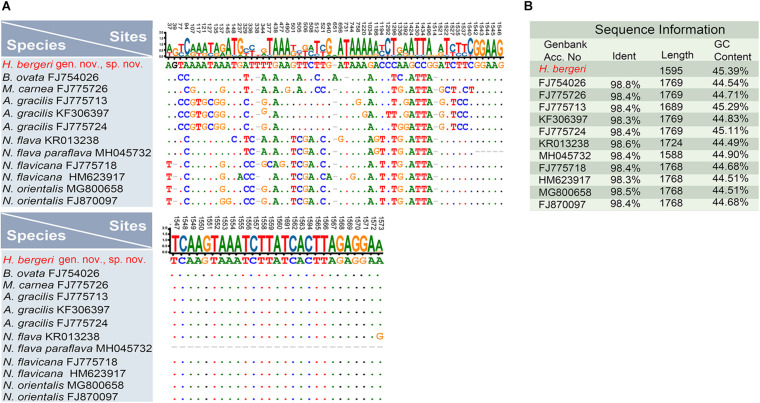
Nucleotide differences and similarities between *Heterobakuella bergeri* gen. nov., sp. nov. (indicated in red) and other related species based on 18S rRNA gene sequences. **(A)** Positions of unmatched nucleotides; short lines (–) indicate the missing nucleotides and solid dots represent matched sites. **(B)** Sequence information including identity, length, and GC content.

For further comparison of the 18S rRNA gene sequences among *Heterobakuella bergeri* gen. nov., sp. nov. and other seven phylogenetically related species, the program BioEdit v.7.0 (Hall, 1999) was used to calculate the number of unmatched nucleotides and the pairwise identities, using “sequence difference count matrix” and “sequence identity matrix” options, respectively. After manually removing identical nucleotides, a nucleotide matrix with 84 unmatched sites is shown in Figure 7.

## Results

### Zoobank Registration

Present work: urn:lsid:zoobank.org:pub:9F118F25-20DD-4D16-99BA-558491E04EA4.

*Heterobakuella* gen. nov.: urn:lsid:zoobank.org:act:AD8318 E6-5248-442B-8FCF-01344F366E89.

*Heterobakuella bergeri* gen. nov., sp. nov.: urn:lsid:zoobank. org:act:51DBC371-A4C0-42A9-99DF-EDE5779A3F7.

*Anteholosticha perezuzae* sp. nov.: urn:lsid:zoobank.org: act:51DBC371-A4C0-42A9-99DF-EDE5779A3F71.

### Taxonomy and Morphological Description of *Heterobakuella bergeri* gen. nov., sp. nov.

Subclass Hypotrichia Stein, 1859

Order Urostylida Jankowski, 1979

Genus *Heterobakuella* gen. nov.

*Diagnosis.* Urostylids with three clearly differentiated frontal cirri, one buccal and one parabuccal cirrus. Midventral complex composed of cirral pairs and one cirral row. One left and more than one right marginal cirral rows. Transverse and pretransverse cirri present. Caudal and frontoterminal cirri lacking.

*Etymology.* Composite of the Greek prefix hetero + (different) and the generic name *Bakuella*. This alludes to the fact that the new genus is different from the “core bakuellid genus” *Bakuella.*

*Type species. Heterobakuella bergeri* sp. nov.

*Species assignable.* The type species only.

*Heterobakuella bergeri* gen. nov., sp. nov.

*Diagnosis.* Size usually 90–130 × 30–45 μm *in vivo*. Body outline long-ellipsoidal and usually with the anterior part wider than the posterior one. Cortical granules yellow-brown, arranged along cirral rows and dorsal kineties. Adoral zone composed of about 25 membranelles. Seven to eleven transverse cirri, two pretransverse cirri, seven to twelve midventral cirral pairs, and a single cirral row composed of four to eleven cirri. One left and two right marginal cirral rows. Three complete dorsal kineties. About 29 macronuclear nodules. Freshwater habitat.

*Dedication.* We dedicate this new species to our eminent colleague, Prof. Helmut Berger (Consulting Engineering Office for Ecology, Salzburg, Austria) in recognition of his contributions to ciliatology.

*Type locality.* Lake Weishan (34°46′14″N, 117°12′56″E), Shandong Province, China.

*Material deposited.* A slide (No. SWY2019111201-1) with the protargol-impregnated holotype specimen and two slides (No. SWY2019111201-2, 3) with protargol-impregnated paratypes are deposited in the Laboratory of Protozoology, Ocean University of China.

*Description*. Cell size usually 90–130 × 30–45 μm, body length to width ratio about 2.5–3.5:1 *in vivo*, i.e., generally shorter and narrower than in protargol preparations (104–158 × 31–77 μm) due to the bleaching procedure (Table 1). Body rather flexible but not contractile, outline elongate elliptical to elongate ovoid, usually with anterior part wider than the posterior one (Figures 2A–D, 3A,B). Cytoplasm colorless, contains numerous colorless globules (about 0.4–0.8 μm in diameter) (Figures 2E, 3E,F), food vacuoles (about 5–25 μm across) in mid-body and posterior region, containing bacteria or algae (Figures 2A,B, 3B). Cortical granules yellow-brown, globular, about 0.5–1.3 μm in diameter, sparsely arranged along cirral rows and dorsal kineties (Figures 2C–E, 3E,F). Contractile vacuole about 11–15 μm across in diastole, located anterior to equator about two-fifths of body length near left margin (Figures 2A,B, 3A,B). Approximately 29 (22–46) irregularly ellipsoidal to ovoidal macronuclear nodules, 8–16 × 3–6 μm in size after protargol impregnation, scattered throughout cytoplasm (Figures 2G, 3D,G–I); one or two globular micronuclei, 2–5 μm in diameter, attached to macronuclear nodules (Figures 2G, 3H). Locomotion by slow to moderately rapid crawling on bottom of Petri dish, or by swimming while rotating about longitudinal axis.

**TABLE 1:** Morphometric characterizations of *Heterobakuella bergeri* gen. nov., sp. nov. (upper line) and *Anteholosticha perezuzae* sp. nov. (lower line).

**Character^a^**	**Min**	**Max**	**Mean**	**M**	**SD**	**CV**	***n***
Body, length	104	158	130.1	131	12.3	9.4	26
	135	275	204.1	202.5	34.1	16.7	16
Body, width	31	77	58.8	60	11.3	18.9	24
	20	35	39.7	30.0	4.6	15.6	16
Body length:width ratio	1.7	4.3	2.3	2.2	0.6	26.9	24
	4.9	9.3	7.0	7.0	1.3	18.9	16
Adoral zone, length	27	72	40.6	39	9.8	25.2	26
	25	40	35.0	35.0	4.1	11.7	16
Adoral zone,% of body length	24	53	31	30	6.7	22.6	26
	13	22	17	18	2.4	13.9	16
Adoral membranelles, number	20	29	24.4	24.5	2.5	10.0	20
	19	26	22.8	22.5	1.7	7.4	16
Frontal cirri, number	3	3	3.0	3.0	0	0	20
	3	3	3.0	3.0	0	0	16
Frontoterminal cirri, number	0	0	0	0	0	0	16
	2	2	2	2.0	0	0	16
Buccal cirrus, number	1	1	1.0	1.0	0	0	20
	1	1	1.0	1.0	0	0	16
Midventral cirral pairs, number	7	12	10.0	10.5	1.6	14.9	20
	17	31	24.3	25.0	3.8	15.4	16
Cirri in midventral cirral row, number	4	11	7.4	7.0	1.8	25.7	20
	0	0	0	0	0	0	25
Pretransverse cirri, number	2	2	2.0	2.0	0	0	20
	1	2	1.7	2.0	0.5	28.4	16
Transverse cirri, number	7	11	9.0	9.0	1.2	13.1	15
	4	7	5.1	5.0	1.0	19.7	16
Left marginal cirral row, number	1	1	1.0	1.0	0	0	20
	1	1	1.0	1.0	0	0	16
Right marginal cirral row, number	2	2	2.0	2.0	0	0	20
	1	1	1.0	1.0	0	0	16
Cirri in left marginal cirral row, number	23	36	28.5	27.5	3.8	13.9	20
	31	62	47.3	48.0	8.2	17.4	16
Cirri in right marginal cirral row 1, number	15	34	26.6	26	5.1	19.4	20
	38	61	49.4	49.5	5.8	11.7	16
Cirri in right marginal cirral row 2, number	27	40	34.3	35	3.9	11.1	20
	0	0	0	0	0	0	20
Dorsal kineties, number	3	3	3	3.0	0	0	10
	3	3	3.0	3.0	0	0	16
Dikinetids in dorsal kinety 1, number	10	18	13.4	14	2.6	18.4	10
	11	21	15.0	15	2.6	17.6	9
Dikinetids in dorsal kinety 2, number	14	20	16.4	16	1.7	10.5	10
	14	23	18.6	18	2.9	15.7	9
Dikinetids in dorsal kinety 3, number	14	23	19.1	20	3.0	15.2	10
	13	21	17.3	18	2.4	13.8	9
Macronuclear nodules, number	22	46	29.1	28.5	5.6	19.7	22
	24	73	57.6	59.5	12.2	21.1	16
Micronuclei, number	1	2	1.7	2.0	0.4	22.3	22
	*	*	*	*	*	*	*

*^*a*^Based on protargol-impregnated specimens. All linear measurements in μm.*

“*” represents unavailable data. CV, coefficient of variation in %; Max, maximum; Mean, arithmetic mean; M, median; Min, minimum; n, number of cells measured; SD, standard deviation.

Adoral zone continuous, extending to about 33% (31–38%) of body length *in vivo*, 31% (24–43%) after protargol impregnation, composed of 20–29 membranelles, membranelle cilia 6–13 μm long. Paroral and endoral membranes nearly equal in length, both slightly curved, optically intersect at posterior 33% of paroral (Figures 2F, 3C,G). Most cirri about 10–12 μm long *in vivo* except frontal and transverse cirri which are 13–14 μm long, 14–17 μm long, respectively. Three clearly differentiated frontal cirri, rearmost one adjacent to distal end of adoral zone (Figures 2F, 3J). Single parabuccal cirrus behind rightmost frontal cirrus (Figure 2F). Single buccal cirrus situated at level of anterior 33% of paroral membrane (Figures 2F, 3J). Midventral complex extends nearly to pretransverse cirri, anterior part composed of seven to twelve midventral cirral pairs, arranged in typical zig-zag pattern, posterior part composed of four to eleven unpaired ventral cirri in a single row (Figures 2F, 3G,H,K). Seven to eleven transverse cirri in J-shaped pattern, two pretransverse cirri (Figures 2F, 3G,H,L). Consistently one left marginal cirral row (LMR) with 23–36 cirri, two right marginal cirral rows (RMR), rightmost with 27–40 cirri, the more medial one with 15–34 cirri (Figure 2F); anterior portions of both RMR usually extend onto dorsal side (Figures 2F,G). Three complete dorsal kineties, two or three additional dorsal bristles to right of anterior end of dorsal kinety 3 (Figures 2G, 3I). Caudal and frontoterminal cirri absent.

### Taxonomy and Morphological Description of *Anteholosticha perezuzae* sp. nov.

Genus *Anteholosticha* Berger, 2003

*Anteholosticha perezuzae* sp. nov.

*Diagnosis.* Body size 140–260 × 25–40 μm *in vivo*. Body outline vermiform, length:width ratio on average 7 (5–9):1 after protargol impregnation. Cortical granules biconcave, greenish, about 1.0–1.5 μm in diameter. Adoral zone continuous, with about 23 membranelles. Three frontal, two frontoterminal, three to six transverse, one or two pretransverse cirri. About 24 midventral cirral pairs. Three complete dorsal kineties. About 58 macronuclear nodules. Brackish water habitat.

*Dedication.* We dedicate this new species to our esteemed colleague, Prof. Blanca Pérez-Uz (Department of Genetics, Physiology and Microbiology, Faculty of Biological Sciences, Universidad Complutense de Madrid, Spain) in recognition of her contributions to protistology.

*Type locality.* A brackish stream near Tangdao Bay, Qingdao, China (35°56′18″N, 120°12′44″E).

*Material deposited.* A slide (No. ZTY2017032701-1) with the protargol-impregnated holotype and five slides (No. ZTY2017032701-2–6) with protargol-impregnated paratypes are deposited in the Laboratory of Protozoology, Ocean University of China.

*Description*. Size about 140–260 × 25–40 μm *in vivo*, usually 200 × 30 μm, after protargol impregnation. Body vermiform, length:width ratio about 5–9:1 (Figures 4A–D,L–N); dorsoventrally flatted about 3:2; flexible but not contractile. Contractile vacuole approximately 8–12 μm across in diastole, located at about anterior 33–43% of body length (Figures 4A–D,L–N, arrowheads). Body brownish at low magnification (Figures 4L–N). Cytoplasm colorless, packed with ellipsoidal structures, probably mitochondria (Figures 4G, 5C, about 3 or 4 μm in diameter). Food vacuoles not observed. Cortex flexible, contains biconcave shaped, greenish cortical granules, about 1.0–1.5 μm in diameter, evenly arranged in a line along every cirral row and dorsal kinety (Figures 4E–G, 5A–C). About 24–73 (on average 58) irregularly ellipsoidal macronuclear nodules scattered throughout cytoplasm, individual nodules approximately 5–10 × 3–5 μm in size after protargol impregnation; micronuclei difficult to determine because hardly distinguishable from similar-sized and similarly impregnated cytoplasmic inclusions (Figures 4K, 5I). Crawls moderately slowly on debris particles, sometimes swims by rotation about main body axis.

Buccal field distinctly narrow, extending from 13 to 22% of body length (Figures 4A–D,J,L–N, 5I). Adoral zone continuous, with 19–26 membranelles (Figures 4H,J, 5D,I); paroral and endoral membranes almost straight, partially overlap (Figures 4H,J, 5D); paroral membrane begins anterior to endoral, slightly shorter than endoral, 10–16 μm long in protargol preparations, endoral membrane about 15–19 μm long after protargol impregnation (Figures 4H, 5D). Three clearly differentiated frontal cirri (Figures 4H,J, 5D), approximately 15–18 μm long *in vivo*. Single parabuccal cirrus, about 15 μm long *in vivo*, behind rightmost frontal cirrus to left of frontoterminal cirri (Figures 4I, 5D). One buccal cirrus, about 10 μm long *in vivo*, situated right of mid-portion of paroral membrane (Figures 4H,J, 5D). Two frontoterminal cirri, located right of parabuccal cirrus (Figures 4H,J, 5D). Midventral complex consists of 17–31 midventral cirral pairs arranged in a zig-zag pattern (Figures 4K, 5H). One or two pretransverse cirrus/cirri anterior to transverse cirri (Figures 4I,J, 5F,G). Cilia of frontoterminal, midventral cirral, and pretransverse cirri about 8–13 μm long. Three to six transverse cirri, about 13–17 μm long *in vivo*, in oblique or slightly “J” shaped row (Figures 4I,J, 5F,G). One left and one right marginal cirral row, composed of 31–62, 38–61 cirri, respectively, cirri usually 10–14 μm long, sometimes up to 17 μm long after protargol preparations (Figures 4J,K, 5E–G,I). Dorsal bristles about 3–4 μm long *in vivo*, arranged in three bipolar rows; dorsal kinety 1 composed of 11–21 dikinetids, kinety 2 of 14–23 dikinetids, and kinety 3 of 13–21 dikinetids (Figures 4K, 5E; Table 1). Two additional dorsal bristles (dikinetids) at anterior end of the right marginal cirral row (Figures 4K, 5E). Caudal cirri absent.

### Phylogenetic Analyses Based on 18S rRNA Gene Sequences

The GenBank accession number, length, and G + C content of the 18S rRNA gene sequences of the two species are as follows: *Heterobakuella bergeri* gen. nov., sp. nov., MW692986, 1,595 bp, 45.39%; *Anteholosticha perezuzae* sp. nov., MW692987, 1,618 bp, 48.75%. Topologies of the maximum likelihood (ML) and Bayesian inference (BI) trees are nearly congruent; thus, only the ML tree is shown with support values from both algorithms (Figure 6). In Figure 6, *Heterobakuella bergeri* gen. nov., sp. nov. and *Anteholosticha perezuzae* sp. nov. are included in the “core urostylids” with strong statistical support.

In the 18S rRNA gene tree, *Heterobakuella bergeri* gen. nov., sp. nov. falls within a group including *Bergeriella ovata*, *Monocoronella carnea*, *Anteholosticha gracilis*, and *Neourostylopsis* spp. with full support. However, the position of *H. bergeri* gen. nov., sp. nov. within this well-supported clade is not robust, as without support in the ML tree (44%) and having an incongruent topology in the BI tree. In the ML tree, *H. bergeri* gen. nov., sp. nov. is sister to a cluster comprising *B. ovata*, *M. carnea*, and *A. gracilis* without support (44%). This poorly resolved cluster is then sister to the strongly supported clade (97%) including *Neourostylopsis* species with full support. While in the BI analysis, *H. bergeri* gen. nov., sp. nov. is placed as a polytomy with *B. ovata*, *M. carnea*, and a clade comprising *A. gracilis*, *Neourostylopsis* spp., respectively (insert in Figure 6).

Sequence comparisons between *H. bergeri* gen. nov., sp. nov. and these molecular related species show that *H. bergeri* gen. nov., sp. nov. differs from *B. ovata* by 18 nucleotide positions (98.8% sequence identity), from *M. carnea* by 25 nucleotide positions (98.4% sequence identity), from *A. gracilis* (three populations) by 26 or 27 nucleotide positions (98.3% or 98.4% sequence identity), from *N. flava* by 24 nucleotide positions (98.6% sequence identity), from *N. flava paraflava* by 21 nucleotide positions (98.4% sequence identity), from *N. flavicana* (two populations) by 26 or 27 nucleotide positions (98.4% or 98.3% sequence identity), and from *N. orientalis* (two populations) by 24 or 25 nucleotide positions (98.4% or 98.5% sequence identity), respectively (Figure 7).

*Anteholosticha perezuzae* sp. nov. does not cluster with any congeners in ML and BI analysis, and clusters with *Apourostylopsis sinica* (Shao et al., 2008) Chen et al., 2013 (EU220227) without support in ML analysis (46%) and with strong support in BI analysis (0.98). This sister cluster groups with a clade comprising two *Metaurostylopsis* species (*M. struederkypkeae* and *M. antarctica*) with weak support in ML analysis (55%) and high support in BI analysis (0.98). Then, the forming sister group among them clusters together with another clade comprising *Thigmokeronopsis* spp. and two populations of *Apokeronopsis ovalis.* Other *Anteholosticha* species are scattered throughout the tree as shown in Figure 6, again indicating the non-monophyly of *Anteholosticha.*

## Discussion

### Comparison of *Heterobakuella bergeri* gen. nov., sp. nov. With Similar Taxa and Basis for the Erection of the Genus

**FIGURE 8:**
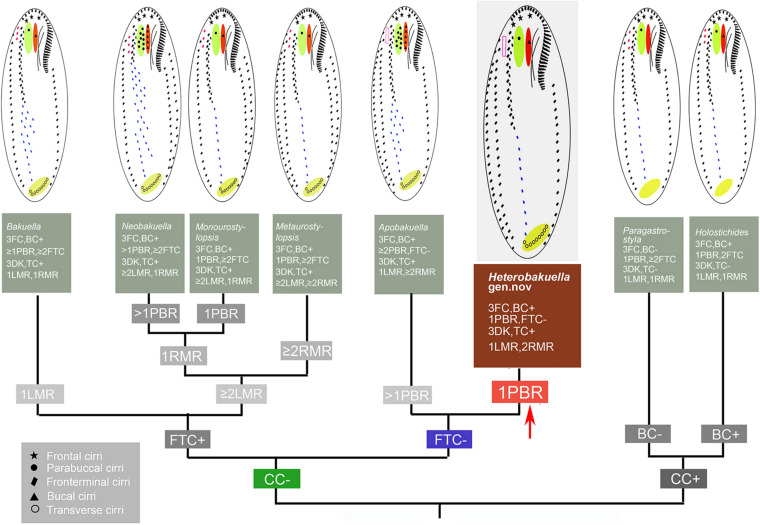
Comparisons of morphologically related genera. BC, buccal cirri; CC, caudal cirri; DK, dorsal kineties; FC, frontal cirri; FTC, frontoterminal cirri; LMR, left marginal cirral row; PBR, parabuccal row; RMR, right marginal cirral row; TC, transverse cirri.

Seven urostylid genera with a continuous adoral zone, three frontal cirri, and midventral complex composed of cirral pairs and row(s) should be compared with *Heterobakuella* gen. nov., namely, *Apobakuella*, *Bakuella*, *Holostichides*, *Metaurostylopsis*, *Monourostylopsis* Song et al., 2020, *Neobakuella*, and *Paragastrostyla* (Berger, 2006; Li et al., 2011; Jiang et al., 2013; Song et al., 2020; Figure 8). Of these, *Apobakuella* is most similar to *Heterobakuella* gen. nov. in terms of the cirral pattern. Hitherto, *Apobakuella*, with the type species *A. fusca*, is monotypic (Jiang et al., 2013). Morphologically, *Heterobakuella bergeri* gen. nov., sp. nov. can be distinguished from *A. fusca* by the smaller body size (90–130 × 35–45 μm vs. 150–210 × 50–60 μm), cortical granules (one type vs. two types), buccal cirri (one vs. three to nine), parabuccal cirri (one vs. five to nine arranged in two or three rows), fewer midventral cirral rows with unpaired cirri in posterior portion(s) (one vs. four to nine), and habitat (freshwater vs. brackish). In addition, *Heterobakuella* gen. nov. can be easily separated from *Holostichides* and *Paragastrostyla* by transverse cirri (present vs. absent in *Holostichides* and *Paragastrostyla*) (Berger, 2006; Zhu et al., 2019), and from the other four genera by marginal cirral rows (a single left and two right rows vs. a single marginal cirral row on each side in *Bakuella*; two or more marginal cirral rows on each side in *Metaurostylopsis*; more than one left and single right marginal cirral rows in *Monourostylopsis* and *Neobakuella*), frontoterminal cirri (absent vs. present in *Bakuella*, *Metaurostylopsis*, *Monourostylopsis*, and *Neobakuella*) (Li et al., 2011; Jo et al., 2015; Lu et al., 2016; Moon et al., 2020; Song et al., 2020; Zhang et al., 2020b). Generic classification of hypotrichs is traditionally based on characteristics of the ciliature and nuclear apparatus in morphostatic and dividing cells (for reviews, see Berger, 1999, 2006, 2008, 2011). Our new species distinctly differs from the morphologically most similar genus, *Apobakuella*, as mentioned previously. In addition, the phylogenetic trees based on 18S rRNA gene sequences show our new species is distant from *A. fusca*, the sequences of these two species differing in 68 nucleotide positions. Thus, because our new species cannot be assigned to any existing genus, we erect the new genus, *Heterobakuella.*

### Familial Classification of *Heterobakuella* gen. nov.

Although Lynn (2008) merged all genera within Bakuellidae and most genera of two other families (e.g., Holostichidae and Urostylidae) from Berger’s (2006) classification into a single large family, Urostylidae, we prefer Berger’s (2006) classification since all genera assigned to Urostylidae sensu Lynn (2008) are paraphyletic and require further study and possibly further subdivision. The monophyly of Bakuellidae has recently been challenged in a number of reports (Lynn, 2008; Yi et al., 2008a,b; Chen et al., 2011; Dai and Xu, 2011; Foissner, 2016; Lyu et al., 2018), and additional data are needed to better define the family. *Heterobakuella bergeri* gen. nov., sp. nov. has morphological characteristics typical for Bakuellidae, including three frontal cirri and a midventral complex composed of pairs and row(s) of unpaired cirri (Berger, 2006). However, *H. bergeri* gen. nov., sp. nov. is distant from the well-known bakuellids including *Apobakuella*, *Bakuella*, *Diaxonella*, and *Neobakuella* in the 18S rRNA gene tree. Morphogenetic features of *H. bergeri* gen. nov., sp. nov. are, as yet, unknown. For these reasons, a confident familial assignment of *Heterobakuella* cannot be made at the current state of knowledge and we consider the genus as incertae sedis in Urostyloidea.

### Comparison of *Anteholosticha perezuzae* sp. nov. With Congeners

*Anteholosticha perezuzae* sp. nov. matches the generic definition of *Anteholosticha* given by Berger (2003). Hitherto, *Anteholosticha* is non-monophyletic and comprises more than 40 species (Berger, 2003, 2006, 2008; Li et al., 2011; Xu et al., 2011; Park et al., 2013; Fan et al., 2016; Chen et al., 2018, 2020; Jung et al., 2021). In terms of the vermiform shape (length:width ratio more than 5:1), the presence of many (more than 20) macronuclear nodules, three enlarged frontal cirri, three dorsal kineties, and a continuous adoral zone of membranelles, the following three species, namely *A. fasciola* (Kahl, 1932) Berger, 2003, *A. grisea* (Kahl, 1932) Berger, 2003, and *A. violacea* (Kahl, 1932) Berger, 2003, should be compared with *Anteholosticha perezuzae* sp. nov. (Berger, 2003, 2006).

*Anteholosticha fasciola* (Kahl, 1932) requires redescription (for a review, see Berger, 2006, page 441) but can be easily distinguished from *A. perezuzae* sp. nov. by shape (length:width ratio up to 10:1 vs. wider shape with the length:width ratio 5–9:1) and buccal cirri (two enlarged, possibly buccal cirri vs. one buccal cirrus). Li et al. (2009) classified a Chinese population as *A. fasciola* and transferred it to a new genus, *Nothoholosticha* Li et al., 2009, which is mainly diagnosed by an atypical bicorona in which the anterior corona is usually formed by four frontal cirri. We compared the Chinese population with the type population described by Kahl (1932) and found that they are likely two different species based on the body shape, the cirri along undulating membranes, and frontal cirri. Specifically, compared with the Chinese population, Kahl’s (1932) population displays a more slender shape with the length:width ratio 10:1 (vs. about 6:1), possesses two enlarged, possibly buccal cirri to the right of the undulating membranes (vs. one buccal cirrus), and three enlarged frontal cirri (vs. four anterior frontal cirri and two posterior cirri) (for a review, see Berger, 2006, page 441). Therefore, we consider the Chinese population of Li et al. (2009) to represent a different species from *A. fasciola* (Kahl, 1932) Berger, 2003. In any event, *Notoholosticha* spp. are easily distinguished from *A. perezuzae* by frontal cirri (six vs. three), atypical bicorona (present vs. absent), adoral zone of membranelles (bipartite vs. continuous).

*Anteholosticha perezuzae* sp. nov. can be distinguished from *A. grisea* (Kahl, 1932) Berger, 2003, because the new species possesses conspicuous greenish cortical granules (about 1.0–1.5 μm in diameter) along the marginal cirral rows, while cortical granules were not mentioned in any population of *A. grisea* (Berger, 2006, page 332). As noted by Berger (2006), Kahl was a very good observer, thus we prefer to accept cortical granules are lacking. *Anteholosticha grisea* invariably displays a blackish body color due to food vacuoles containing ingested rhodobacteria (Berger, 2006, page 332), while *A. perezuzae* sp. nov. shows a brownish body at low magnifications. In addition, *A. grisea* is more likely confined to the freshwater sapropel, while *A. perezuzae* sp. nov. is from a brackish stream.

According to Berger (2006), the taxonomy of *Anteholosticha violacea* is rather complicated. At least six populations were reported and the possibility that they belong to different species could not be excluded, thus Berger simply accepted Kahl’s data (Kahl, 1928, 1932; Berger, 2006). *Anteholosticha perezuzae* sp. nov. differs distinctly from Kahl’s (1928) population by frontal cirri (three enlarged frontal cirri vs. six to seven) and from Kahl’s (1932) second population by dorsal bristles (3–4 μm long vs. at least 8 μm long).

### Phylogenetic Analyses of the Genus *Heterobakuella* and *Anteholosticha*

Considering the position and the specialized pattern of ventral cirri, viz., three frontal cirri and a midventral complex composed of cirral pairs and row(s), *Heterobakuella* might be closely related to the bakuellid taxa, especially to the morphologically similar genus *Apobakuella*. However, a close phylogenetic relationship between *Heterobakuella* and bakuellid taxa is not detected in the very conservative 18S rRNA gene tree, as these taxa do not form a monophyletic assemblage, and *Heterobakuella* branches off earlier than bakuellid taxa. Thus, their similar ventral cirral pattern likely represents a convergently evolved character, viz., the ventral ciliary pattern is analogous and not homologous. On the other hand, *Heterobakuella* groups together with *Bergeriella*, *Monocoronella*, *Anteholosticha gracilis*, and *Neourostylopsis* with the full support in 18S rRNA gene tree (Figure 6). Within this clade, *Bergeriella*, *Heterobakuella*, and *Monocoronella* are three monotypic genera with evolutionary novelties, and type species of which are *B. ovata*, *H. bergeri*, and *M. carnea*, respectively. Morphologically, *H. bergeri* can be easily distinguished from *B. ovata* by frontal cirri (three vs. 6–13) and postoral ventral rows (absent vs. present) (Liu et al., 2010), from *M. carnea* by frontal cirri arranged in monocorona (absent vs. present) (Chen et al., 2011), and from *A. gracilis* and *Neourostylopsis* spp. by midventral complex composed anteriorly of pairs and posteriorly of unpaired cirri (present vs. absent) (Berger, 2006; Chen et al., 2013). Thus, to understand their evolutionary relationship, more discoveries of further congeners and more genes must be awaited.

Because the genus *Anteholosticha* was established based only on a combination of plesiomorphies in the absence of obvious synapomorphies, Berger (2003, 2006) had hypothesized the non-monophyly of *Anteholosticha*. Subsequently, the non-monophyly of *Anteholosticha* has been reported in many previous studies (Park et al., 2013; Zhao et al., 2015; Fan et al., 2016; Chen et al., 2018, 2020; Paiva, 2020; Jung et al., 2021). Our analyses also confirm that *Anteholosticha* species are scattered among different branches inside the core urostylid assemblage. Morphological and molecular data for the newly discovered species *A. perezuzae* sp. nov. is highly incongruent. *Anteholosticha perezuzae* clusters with *Apourostylopsis sinica* (EU220227) without support in ML analysis and with strong support in BI analysis (0.98). However, morphologically, *A. perezuzae* sp. nov. can be easily separated from *A. sinica* by body size (140–260 × 25–40 μm vs. 100–120 × 30 um *in vivo*), one type of cortical granules (vs. both two), number of midventral cirral pairs (17–31 vs. 11–17), number of left marginal cirral rows (one vs. three), and number of left marginal cirral rows (one vs. two) (Shao et al., 2008; Chen et al., 2013). Likewise, if the “good” apomorphies for inferring their evolutionary relationship cannot be found, this polyphyly might be better resolved with future analysis of additional taxa. Regarding this problem of taxonomic and phylogenetic resolution, a new classification system proposed by Paiva (2020) might give us some inspirations. According to Paiva (2020), the newly established taxon Kentrurostylida corresponds to the “core urostylids”. Within the taxon, two secondary taxa Simplicitergida and Hispidotergida were established, based on three dorsal bristle rows and numerous dorsal bristles, respectively. Accordingly, *A. antecirrata*, *A. gracilis*, *A. manca*, *A. multicirriata*, and *A. paramanca* were assigned to Simplicitergida, while the other species, *A.* cf. *azerbaijanica*, *A. foissneri*, *A. marimonilata*, *A. monilata*, *A. pseudomonilata*, *A. pulchra*, *A. songi*, *A. randani*, and *A. rectangula* were placed in Hispidotergida. Under Paiva’s (2020) classification, *Anteholosticha perezuzae* would be assigned to Simplicitergida. From the current work, it is obvious that the taxonomy and phylogeny of both bakuellid-like taxa and those species currently included in *Anteholosticha* remains in a state of flux.

## Data Availability Statement

The datasets presented in this study can be found in online repositories. The names of the repository/repositories and accession number(s) can be found in the article/supplementary material.

## Author Contributions

YW designed and supervised the research study. WS, TZ, and JD drafted the manuscript. XL and WB revised and improved the manuscript. WS and TZ collected samples and performed staining. All authors contributed to the manuscript and approved the submitted version.

## Conflict of Interest

The authors declare that the research was conducted in the absence of any commercial or financial relationships that could be construed as a potential conflict of interest.

## Publisher’s Note

All claims expressed in this article are solely those of the authors and do not necessarily represent those of their affiliated organizations, or those of the publisher, the editors and the reviewers. Any product that may be evaluated in this article, or claim that may be made by its manufacturer, is not guaranteed or endorsed by the publisher.

## Footnotes

1
https://www.ncbi.nlm.nih.gov/

2
http://guidance.tau.ac.il/

3
http://www.phylo.org/sub_sections/portal/
